# Targeted Therapy for Biliary Tract Cancer

**DOI:** 10.3390/cancers3022243

**Published:** 2011-05-03

**Authors:** Junji Furuse, Takuji Okusaka

**Affiliations:** 1 Department of Internal Medicine, Medical Oncology, Kyorin University School of Medicine, 6-20-2, Shinkawa, Mitaka, Tokyo, 181-8611, Japan; 2 Department of Hepatobiliary and Pancreatic Oncology, National Cancer Center Hospital, 5-1-1 Tsukiji, Chuo-ku, Tokyo 104-0045, Japan; E-Mail: tokusaka@ncc.go.jp

**Keywords:** biliary tract cancer, chemotherapy, molecular targeted agent

## Abstract

It is necessary to establish effective chemotherapy to improve the survival of patients with biliary tract cancer, because most of these patients are unsuitable candidates for surgery, and even patients undergoing curative surgery often have recurrence. Recently, the combination of cisplatin plus gemcitabine was reported to show survival benefits over gemcitabine alone in randomized clinical trials conducted in the United Kingdom and Japan. Thus, the combination of cisplatin plus gemcitabine is now recognized as the standard therapy for unresectable biliary tract cancer. One of the next issues that need to be addressed is whether molecular targeted agents might also be effective against biliary tract cancer. Although some targeted agents have been investigated as monotherapy for first-line chemotherapy, none were found to exert satisfactory efficacy. On the other hand, monoclonal antibodies such as bevacizumab and cetuximab have also been investigated in combination with a gemcitabine-based regimen and have been demonstrated to show promising activity. Furthermore, clinical trials using new targeted agents for biliary tract cancer are also proposed. This cancer is a relatively rare and heterogeneous tumor consisting of cholangiocarcinoma and gallbladder carcinoma. Therefore, a large randomized clinical trial is necessary to confirm the efficacy of chemotherapy, and international collaboration is important.

## 1. Introduction

Biliary tract cancer is rather common in Latin America and Asia, including Japan, while being relatively rare in European countries and the United States; approximately 16,000 patients in Japan and 5,000 patients in the United States are newly diagnosed as having this cancer each year [1-3]. The varied geographic distribution of the risk factors for biliary tract cancer, including primary sclerosing cholangitis, hepatolithiasis, congenital biliary cystic diseases, chemical agents, and hepatitis virus infections appears to contribute to the differences in the incidence rates among ethnic groups [1-4].

Bile duct cancer is subdivided according to the anatomic location of origin into intrahepatic cholangiocarcinoma, gallbladder cancer, extrahepatic cholangiocarcinoma and ampulla of Vater cancer. Although surgery currently remains the only potentially curative treatment for each of the aforementioned diseases, most patients are diagnosed at an unresectable advanced stage of the disease. While chemotherapy is applicable for all of these diseases, different carcinogenetic pathways and sensitivities to therapies have been demonstrated for each of them. The survival in patients with unresectable biliary tract cancer has been shown to differ by the tumor type, that is, gallbladder carcinoma, intrahepatic cholangiocarcinoma, or extrahepatic cholangiocarcinoma. It would, therefore, be ideal to conduct separate clinical trials in each cancer. However, it is not practical, because each of these biliary tract cancers is relatively rare. For the development of new chemotherapeutic regimens for biliary tract cancer, randomized clinical trials with an appropriate stratification strategy are required, including by the tumor types.

Despite the numerous phase II studies conducted of treatments for advanced biliary tract cancer, no accepted standard treatment for this tumor type has been established yet, because of the low incidence and small number of patients and the lack of adequately powered randomized controlled trials. A number of studies have investigated the usefulness of gemcitabine alone or tegafur/gimeracil/oteracil potassium (S-1) alone, and also gemcitabine-based combination regimens (Table 1) [5-22]. Based on the results of phase II studies, the Japanese guideline for biliary tract and ampullary carcinomas recommends gemcitabine alone or S-1 alone as the first line chemotherapy [23]. Recently, randomized controlled trials comparing the combination of cisplatin plus gemcitabine with gemcitabine alone have shown the survival benefit of the former regimen (Table 1) [24,25]. Furthermore, a randomized controlled trial among best supportive care, fluorouracil plus folinic acid and gemcitabine plus oxaliplatin (Gemox) revealed improved survival with Gemox in patients with unresectable gallbladder cancer as compared with best supportive care and fluorouracil plus folinic acid (Table 2) [26]. Thus, the combination of gemcitabine plus a platinum agent (cisplatin or oxaliplatin) has come to be recognized as standard therapy for unresectable biliary tract cancer.

One of the next issues that needs to be addressed is whether molecular targeted agents might also be effective against biliary tract cancer. Until date, no large clinical trials using targeted agents have been conducted for biliary tract cancer, however, some of these agents appear to offer promise. In this paper, the results of preclinical experiments and clinical trials of molecular targeted therapy for the treatment of biliary tract cancer are reviewed, and the possibilities and future directions of the use of targeted agents are discussed.

## 2. Preclinical Studies of the Molecular Biology of Biliary Tract Cancer

Some growth factors, including epidermal growth factor receptor (EGFR) and vascular endothelial growth factor receptor (VEGFR), and various signal transduction pathways that play important roles in the progression, proliferation and metastasis of various cancers have been identified. Some studies have demonstrated overexpression of EGFR and VEGFR, or mutations of their signaling pathways in biliary tract cancer [27]. Nonomura *et al.* [28] reported overexpression rates by 32.4% of EGFR, by 59.5% of EGF, and by 89.2% of ras p21 in 37 intrahepatic cholangiocarcinomas, with all the rates being statistically significantly different as compared with those in normal tissues. Recently, Yoshikawa *et al.* [29] demonstrated EGFR, VEGF and human epidermal growth factor receptor (HER) 2 overexpression in 27.4, 53.8 and 0.9% cases of intrahepatic cholangiocarcinoma, and in 19.2, 59.2 and 8.5% of cases of extrahepatic cholangiocarcinoma, respectively. They reported the existence of a correlation between the prognosis and EGFR expression, and the survival duration of EGFR-positive patients was significantly longer than that of EGFR–negative patients, both among cases of intrahepatic cholangiocarcinoma and extrahepatic cholangiocarcinoma. Furthermore, VEGF expression has been shown to be associated with intrahepatic metastasis in cases of intrahepatic cholangiocarcinoma [29].

Biliary tract cancer includes various types of cancers, each with different molecular biological characteristics. For example, overexpression of EGFR has been reported to be observed in 10.7%, 5.1%, 12.4% and 0% of cases of intrahepatic cholangiocarcinoma, extrahepatic cholangiocarcinoma, gallbladder cancer and ampulla of Vater cancer, respectively [30]. Furthermore, overexpression of ErbB-2 has been reported in 0%, 5.1%, 15.7% and 11.5% of cases of intrahepatic cholangiocarcinoma, extrahepatic cholangiocarcinoma, gallbladder cancer and ampulla of Vater cancer, respectively [30]. Relationships between the presence /absence of various gene mutations and the efficacy of molecular targeted agents have been identified in various cancers; for example, the efficacy of anti-EGFR antibody was limited to colorectal cancer patients with wild-type KRAS expression in the tumor [31]. There is as yet, however, no consensus on the molecular-biologic characteristics of biliary tract cancer.

Few preclinical studies of molecular targeted agents for biliary tract cancer have been reported. In an examination conducted using human cholangiocarcinoma cell lines, ZD6474, an inhibitor of VEGFR and EGFR signaling, showed promising anticancer activity [32]. This study revealed that the absence of KRAS mutation and presence of EGFR amplification may be potentially predictive molecular markers of the sensitivity of cholangiocarcinoma to EGFR-targeted therapy [32]. Thus, therapeutically beneficial effects of molecular targeted agents against biliary tract cancer are expected.

## 3. Clinical Trials of EGFR Inhibitors for Biliary Tract Cancer

Erlotinib is an orally active, potent, selective inhibitor of EGFR/HER1 tyrosine kinase, and a phase II study of erlotinib for unresectable biliary tract cancer has been reported. The results of this trial (response rate of 17% and median overall survival of 7.5 months) suggested a therapeutic benefit of EGFR blockade with erlotinib in patients with biliary tract cancer (Table 3) [33], however, no further investigation was conducted. Since lapatinib is an oral dual kinase inhibitor of EGFR and Her-2/neu, an antitumor effect of this agent against biliary tract cancer was expected. A phase II study of lapatinib for hepatocellular carcinoma and biliary tract cancer was conducted, however, no response was observed in patients with biliary tract cancer [34]. Thus, monotherapy with anti-EGFR inhibitors as first-line therapy may yield only minimum antitumor activity against biliary tract cancer. Thus, further investigation of anti-EGFR inhibitors does not appear to be warranted.

On the other hand, the effect of cetuximab, an anti-EGFR antibody, administered in combination with the Gemox regimen has also been investigated (Table 3) [35,36]. A phase II study of Gemox plus cetuximab showed promising efficacy, with a response rate of 63% and median overall survival rate of 15.2 months [35]. A randomized phase II study comparing Gemox plus cetuximab and Gemox alone is currently under investigation, and the results of an interim analysis were reported at the American Society of Clinical Oncology meeting in 2010 [36]. In regard to the relationship between KRAS mutation and the efficacy of cetuximab against biliary tract cancer, only 3 of the 30 patients in the phase II study of Gemox plus cetuximab had KRAS mutation in the tumor, with two of the three patients showing partial response and one showing stable disease. Thus, no definite correlation was noted between KRAS mutation and the treatment efficacy [35]. The sample size in this study was small, and further large-scale clinical trials of the combination therapy are needed to further clarify the efficacy and safety of cetuximab. Furthermore, the expression status of various key molecular targets, such as KRAS, B-raf and MEK, should be examined to identify patients with biliary tract cancer who may benefit from treatment with anti-EGFR monoclonal antibody.

## 4. Clinical Trials of Anti-Angiogenic Inhibitors for Biliary Tract Cancer

Vascular endothelial growth factor is related to angiogenesis and is reported as one of the important factors involved in the angiogenesis of various malignancies; VEGFR has been shown to promote the growth and metastasis of various cancers. Tumor vessels are structurally and functionally abnormal, contributing to increase of the interstitial fluid pressure within the tumor [37-39]. Anti-VEGF treatment results in pruning of the tumor vasculature, reduction in vessel tortuosity, and a drop in the interstitial fluid pressure, a process termed as vessel normalization [39]. Furthermore, it has been reported that combined use of a cytotoxic drug with anti-VEGF agent leads to a rapid decrease of the interstitial fluid pressure, which may enhance the delivery of chemotherapeutic agents to tumor cells [39], thereby leading to tumor size reduction and improvement of the survival rates. Various inhibitors targeting VEGF or VEGFR have also been investigated for application to the treatment of biliary tract cancer.

Sorafenib, an oral multikinase inhibitor, blocks tumor cell proliferation mainly by targeting Raf/MAPK-ERK kinase/extracellular signal-regulated kinase signaling at the level of Raf kinase, and exerts an antiangiogenic effect by targeting VEGFR-2/-3. Sorafenib has been reported to exert antitumor effect on renal cell cancer and hepatocellular carcinoma, and also to show survival benefits in patients with these tumors [40,41]. Until date, the potential usefulness of sorafenib for biliary tract cancer has been investigated in two phase II studies, however, scant efficacy was noted in both trials, with a response rate of 2 and 6%, median progression-free survival of 2.0 and 2.3 months, and median overall survival of 4.4 and 6.0 months, respectively, in the two trials [42,43].

Bevacizumab, a recombinant humanized monoclonal antibody directed against VEGF, is an important therapeutic agent with promising effect against several malignancies, including colorectal, lung, breast and renal cell cancers, and has been investigated in phase II studies in combination with other agents for biliary tract cancer [44,45]. The combination of Gemox plus bevacizumab yielded promising results, with a response rate of 40% and median overall survival of 12.7 months [44]. Inhibition of VEGF and EGFR by bevacizumab and erlotinib was tested in a phase II study, and modest efficacy was noted, with a response rate of 12% and median overall survival of 9.9 months [45]. This trial was only preliminary and further investigation is expected.

The role of anti-angiogenic agents in the treatment of biliary tract cancer is still not clear. From the results of these clinical trials, anti-angiogenic agents do not appear to be promising. It is necessary to investigate the possible effect of anti-angiogenic agents against biliary tract cancer by conducting experiments *in vivo* for each agent.

## 5. Perspectives of Molecular Targeted Therapy for Biliary Tract Cancer

There are difficulties in conducting clinical trials for biliary tract cancer including large numbers of patients, as biliary tract cancer is a relatively rare disease, and the high frequency of complications such as obstructive jaundice or cholangitis make it difficult to recruit eligible patients. However, the curative resection rate is only 39.7%, on average, and according to retrospective studies, a half of the patients with unresectable biliary tract cancer receive chemotherapy in Japan [46,47]. Thus, chemotherapy plays an important role in the treatment of biliary tract cancer in clinical practice, and the development of an effective treatment(s) for biliary tract cancer is urgently needed. Various regimens containing molecular targeted agents are currently under investigation (Table 4).

As mentioned above, combination therapy with gemcitabine plus cisplatin or oxaliplatin has been established as the standard first-line treatment for biliary tract cancer. The next step is focused on the usage of molecular targeted agents. There are two directions in which targeted agents can be expected to be applied in the treatment of biliary tract cancer. One is combination with standard chemotherapy regimens as first-line therapy. Currently, combined treatment using anti-EGFR antibody with cetuximab or bevacizumab has shown promising results in phase II studies of combination with Gemox. Although a randomized phase II study of cetuximab is currently under way, a large comparison study would be needed. The other is the use of monotherapy with targeted agents as 2^nd^ line chemotherapy. Some preclinical experiences show that VEGFR or EGFR inhibitors administered alone might be effective in the treatment of biliary tract cancer. In many patients with progressive disease receiving first-line chemotherapy with the relatively toxic regimen of cisplatin plus gemcitabine or Gemox, the general condition is poor, and serious cholangitis can easily develop. Less toxic therapy, such as monotherapy with a targeted agent, may be useful in such patients.

Biliary tract cancer, which has a heterogeneous disease background, consists of cholangiocarcinoma, gallbladder carcinoma and ampulla of Vater cancer, each having different biologic features. Molecular targeted therapy should be established based on the biologic features, and it is important to identify the characteristic biologic features of each of the aforementioned types of cancer of the biliary tract. The number of patients with each of the cancer types of the biliary tract is small, and furthermore, patients with particular biologic features may be rare. However, efficient development of targeted therapy should be advanced based on the identification of appropriate biological markers.

## 6. Conclusions

Effective chemotherapy is necessary to improve survival of patients with biliary tract cancer. However, biliary tract cancer is a relatively rare disease compared with other gastrointestinal cancers such as colorectal cancer or gastric cancer. It makes large clinical trials difficult to conduct in a single country. Establishment of a new standard chemotherapy using molecular targeted agents is eager, and collaboration among global clinical trials is important.

## Figures and Tables

**Table 1.** Phase II studies of gemcitabine-based regimen for unresectable biliary tract cancer.

**Regimen**	**n**	**Response Rate**	**Median Progression-Free Survival or Time-to-Progression**	**Median Overall Survival**	**Author (Year)**
Gemcitabine	25	36.0%	-	6.9 mo	Gallardo (2001) [5]
Gemcitabine	32	21.9%	5.6 mo	11.5 mo	Penz (2001) [6]
Gemcitabine	30	30.0%	7.0 mo	14.0 mo	Tsavaris (2004) [7]
Gemcitabine	40	17.5%	2.6 mo	7.6 mo	Okusaka (2006) [8]
Tegafur/gimeracil/oteracil potassium (S-1)	40	35%	3.7 mo	9.4 mo	Furuse (2008) [9]
Gemcitabine/cisplatin	30	37%	4.1 mo	4.6 mo	Doval (2004) [10]
Gemcitabine/cisplatin	40	28%	4.7 mo	8.4 mo	Thongprasert (2005) [11]
Gemcitabine/cisplatin	29	35%	3.0 mo	11.0 mo	Kim (2006) [12]
Gemcitabine/oxaliplatin	33	35.5%	5.7 mo	15.4 mo	André (2004) [13]
Gemcitabine/oxaliplatin	31	26.0%	6.4 mo	11 mo	Harder (2006) [14]
Gemcitabine/oxaliplatin	67	14.9%	3.4 mo	8.8 mo	André (2008) [15]
Gemcitabine/oxaliplatin	40	15.0%	4.2 mo	8.5 mo	Kim (2009) [16]
Gemcitabine/oxaliplatin	43	18.9%	4.8 mo	8.3 mo	Jang (2009) [17]
Gemcitabine/capecitabine	45	31%	7.0 mo	14.0 mo	Knox (2005) [18]
Gemcitabine/capecitabine	45	32%	6.0 mo	14.0 mo	Cho (2005) [19]
Gemcitabine/capecitabine	75	29%	6.2 mo	12.7 mo	Riechelmann (2007) [20]
Gemcitabine/capecitabine	44	25%	7.2 mo	13.2 mo	Koeberle (2008) [21]
Gemcitabine/S-1	35	34.3%	5.9 mo	11.6 mo	Sasaki (2009) [22]

**Table 2.** Randomized clinical trials of gemcitabine-based regimens for unresectable biliary tract cancer.

**Regimen**	**n**	**Response Rate**	**Median Progression-Free Survival or Time-to-Progression**	**Median Overall Survival**	**Author (Year)**
Gemcitabine	206	15.5%	5.0 mo	8.3 mo	Valle (2010) [24]
Gemcitabine/cisplatin	204	26.1%	8.0 mo	11.7 mo	
Gemcitabine	42	11.9%	3.7 mo	7.7 mo	Okusaka (2010) [25]
Gemcitabine/cisplatin	41	19.5%	5.8 mo	11.2 mo	
Best supportive care	27	0	2.8 mo	4.5 mo	Sharma (2010) [26]
Fluorouracil/folinic acid	28	14.3%	3.5 mo	4.6 mo	
Gemcitabine/oxaliplatin	26	30.7%	8.5 mo	9.5 mo	

**Table 3.** Clinical trials of molecular targeted agents for unresectable biliary tract cancer.

**Regimen**	**n**	**Response Rate**	**MedianProgression-Free Survival or Time-to-Progression**	**Median Overall Survival**	**Author (Year)**
Erlotinib	42	7%	2.6 mo	7.5 mo	Philip (2006) [33]
Lapatinib	17	0%	1.8 mo	5.2 mo	Ramananthan (2009) [34]
Gem/oxaliplatin/cetuximab	30	63%	8.8 mo	15.2 mo	Gruenberger (2010) [35]
Gem/oxaliplatin	51	16.7%(n=18)	∼5.0 mo	-	Malka (2009) [36]
Gem/oxaliplatin/cetuximab	50	11.1%(n=18)	∼7.0 mo	-	
Sorafenib	36	6%	2.0 mo	6.0 mo	El-Khoueiry (2007) [42]
Sorafenib	46	2%	2.3 mo	4.4 mo	Bengala (2010) [43]
Gem/oxaliplatin/bevacizumab	35	40%	7.0 mo	12.7 mo	Zhu (2010) [44]
Bevacizumab/erlotinib	53	12%	4.4 mo	9.9 mo	Lubner (2010) [45]

**Table 4.** Currently ongoing clinical trials of molecular targeted agents for biliary tract cancer.

**Agent**	**Study**	**n**	**Primary Endpoint**	**Country**
Gemcitabine/oxaliplatin/sorafenib	Phase I/II	58	Progression-free survival	USA
Gemcitabine/vandetanib or placebo	R-phase II	174	Progression-free survival	Italy
Gemcitabine/oxaliplatin/erlotinib	Phase I	22	Maximum tolerated dose, recommended dose of erlotinib	USA
AZD6244	Phase II	35	Objective response rate	USA
Gemcitabine/oxaliplatin or Gemcitabine/oxaliplatin/cetuximab	R-phase II	100	Progression-free survival	France
Gemcitabine/cisplatin/cediranib or Gemcitabine/cisplatin/placebo	Phase II/III	136	Progression-free survival	UK
Gemcitabine/oxaliplatin or Gemcitabine/oxaliplatin/cetuximab	R-phase II	120	Objective response rate	Taiwan
Folfox/cediranib	Phase II	36	Objective response rate	USA
Gemcitabine/cisplatin/selumetinib	Phase I/II	18	Safety and tolerability, recommended dose of selumetinib	UK
Gemcitabine/cetuximab	Phase II	43	Progression-free survival	Belgium
FOLFOX6/bevacizumab	Phase II	24	Progression-free survival	USA
Gemcitabine/irinotecan/panitumumab	Phase II	45	Progression-free survival	USA
Gemox/panitumumab	Phase II	15	Objective response rate	USA
Gemcitabine/sorafenib or Gemcitabine/placebo	R-phase II	103	Progression-free survival	Germany
Gemcitabine/capecitabine/bevacizumab	Phase II	50	Progression-free survival	USA
Gemcitabine/cisplatin/sorafenib	Phase II	39	Progression-free survival	USA
Trastuzumab	Phase II	32	Objective response rate	USA
Bevacizumab/erlotinib	Phase II	55	Objective response rate	USA
Sunitinib	Phase II	59	Time to progression	Korea
Gemox/erlotinib or Gemox	Phase III	180	Time to progression	Korea
Bortezomib	Phase II	35	Objective response rate	USA
Sorafenib/erlotinib	Phase II	50	Progression-free survival	USA
